# Building a Networked Improvement Community: Lessons in Organizing to Promote Diversity, Equity, and Inclusion in Science, Technology, Engineering, and Mathematics

**DOI:** 10.3389/fpsyg.2021.732347

**Published:** 2021-11-18

**Authors:** Chelsea E. Noble, Marilyn J. Amey, Luis A. Colón, Jacqueline Conroy, Anna De Cheke Qualls, Kamla Deonauth, Jeffrey Franke, Alex Gardner, Bennett Goldberg, Thelma Harding, Gary Harris, Sara Xayarath Hernández, T. Lisa Holland-Berry, Omari Keeles, Barbara A. Knuth, Colleen M. McLinn, Judy Milton, Rudisang Motshubi, C. A. Ogilvie, Rosemary J. Perez, Sarah L. Rodriguez, Nancy Ruggeri, Panos S. Shiakolas, Arnold Woods

**Affiliations:** ^1^Department of Educational Administration, Michigan State University, East Lansing, MI, United States; ^2^Department of Chemistry, University at Buffalo, Buffalo, NY, United States; ^3^Department of Counseling, School and Educational Psychology, University at Buffalo, Buffalo, NY, United States; ^4^The Graduate School, University of Maryland, College Park, College Park, MD, United States; ^5^Graduate School, Howard University, Washington, DC, United States; ^6^Office for Research, Northwestern University, Evanston, IL, United States; ^7^Graduate College, Iowa State University, Ames, IA, United States; ^8^Graduate School, Cornell University, Ithaca, NY, United States; ^9^LINK Research Lab, The University of Texas at Arlington, Arlington, TX, United States; ^10^Poly Prep Country Day School, Brooklyn, NY, United States; ^11^Department of Natural Resources and the Environment, Cornell University, Ithaca, NY, United States; ^12^Graduate School, University of Georgia, Athens, GA, United States; ^13^School of Education, Iowa State University, Ames, IA, United States; ^14^Graduate School, Montana State University, Bozeman, MT, United States; ^15^Center for the Study of Higher and Postsecondary Education, University of Michigan, Ann Arbor, MI, United States; ^16^Department of Higher Education and Learning Technologies, Texas A&M University-Commerce, Commerce, TX, United States; ^17^Searle Center for Advancing Learning and Teaching, Northwestern University, Evanston, IL, United States; ^18^Department of Mechanical and Aerospace Engineering, The University of Texas at Arlington, Arlington, TX, United States

**Keywords:** Networked Improvement Community, diversity, STEM, faculty careers, shared leadership

## Abstract

In 2016, 10 universities launched a Networked Improvement Community (NIC) aimed at increasing the number of scholars from Alliances for Graduate Education and the Professoriate (AGEP) populations entering science, technology, engineering, and mathematics (STEM) faculty careers. NICs bring together stakeholders focused on a common goal to accelerate innovation through structured, ongoing intervention development, implementation, and refinement. We theorized a NIC organizational structure would aid understandings of a complex problem in different contexts and accelerate opportunities to develop and improve interventions to address the problem. A distinctive feature of this NIC is its diverse institutional composition of public and private, predominantly white institutions, a historically Black university, a Hispanic-serving institution, and land grant institutions located across eight states and Washington, DC, United States. NIC members hold different positions within their institutions and have access to varied levers of change. Among the many lessons learned through this community case study, analyzing and addressing failed strategies is as equally important to a healthy NIC as is sharing learning from successful interventions. We initially relied on pre-existing relationships and assumptions about how we would work together, rather than making explicit how the NIC would develop, establish norms, understand common processes, and manage changing relationships. We had varied understandings of the depth of campus differences, sometimes resulting in frustrations about the disparate progress on goals. NIC structures require significant engagement with the group, often more intensive than traditional multi-institution organizational structures. They require time to develop and ongoing maintenance in order to advance the work. We continue to reevaluate our model for leadership, climate, diversity, conflict resolution, engagement, decision-making, roles, and data, leading to increased investment in the success of all NIC institutions. Our NIC has evolved from the traditional NIC model to become the Center for the Integration of Research, Teaching and Learning (CIRTL) AGEP NIC model with five key characteristics: (1) A well-specified aim, (2) An understanding of systems, including a variety of contexts and different organizations, (3) A culture and practice of shared leadership and inclusivity, (4) The use of data reflecting different institutional contexts, and (5) The ability to accelerate infrastructure and interventions. We conclude with recommendations for those considering developing a NIC to promote diversity, equity, and inclusion efforts.

## Introduction

In 2016, 10 research universities in the United States launched a Networked Improvement Community (NIC) through the National Science Foundation’s (NSF) Alliances for Graduate Education and the Professoriate (AGEP) program. The NIC’s goal, in alignment with AGEP’s mission, is to increase the number of scholars from AGEP populations – Black and African Americans, Hispanic and Latinx Americans, and American Indians, Alaska Natives, Native Hawaiians, and Native Pacific Islanders – entering science, technology, engineering, and math (STEM) faculty careers. AGEP populations represent an increasing proportion of the science, engineering, and health academic workforce, rising from 6.4% in 1999 to 8.9% in 2019 (National Center for Science and Engineering Statistics, 2021). However, Black and African American, Hispanic and Latinx, and Native American people comprise approximately one-third of adults 18–64 in the United States, so these populations remain markedly underrepresented in STEM academic roles (National Center for Science and Engineering Statistics, 2021). This disparity persists despite decades of efforts to diversify STEM fields (Leboy and Madden, 2012; Whittaker and Montgomery, 2014).

The purpose of this community case study is to describe and reflect on the establishment and evolution of a NIC in the United States higher education context. In this article, we describe how these 10 universities formed a NIC, adapted the NIC to meet the different campus contexts, and launched structural changes and interventions to promote increased representation of AGEP populations in the STEM professoriate. Further, we offer lessons and insights from our work as a NIC, with particular attention to equity, diversity, and inclusion within the NIC.

## What Is a Networked Improvement Community?

Popularized by the Carnegie Foundation, a NIC creates a highly structured learning and design community (Bryk et al., 2010, 2015). This organizational approach brings together stakeholders focused on a well-specified common goal, deep understanding of the problem, and opportunities for change (Bryk et al., 2010). It leverages the power of improvement science and networks to accelerate innovation and improvement through structured, ongoing intervention development, implementation, and refinement (LeMahieu et al., 2017). In higher education, where professional silos frequently result in divisions between stakeholders (Kezar, 2005; Torres and Renn, 2021), NIC structures also function as “an attempt to redefine professional roles and identities as well as the relationships between these stakeholders” (LeMahieu et al., 2017, p. 24).

Feygin et al.’s (2020) metastudy found seven NICs that were studied in K-12 and undergraduate education, with no studies at the graduate level. The authors found “challenges to NIC implementation, such as inconsistent application of Plan Do Study Act (PDSA) cycles, frustration with an onerous process, and burden on teachers and principals” (p. 8) and “NICs are complex organizations that are difficult to implement” (p. 10). As NICs are a newer formalized organizational structure, especially in higher education, there is much to learn about them in practice (LeMahieu et al., 2017). Given their potential for system change, we argue that NIC structures offer great promise to the national effort to broaden representation in STEM.

## Introducing Our Networked Improvement Community

Established in 2016, our NIC was funded through NSF’s AGEP program. In alignment with AGEP’s mission, the goal of our NIC is to increase scholars’ aspirations and persistence in STEM faculty careers primarily by improving campus climate. Efforts to increase compositional diversity and promote inclusion within STEM fields are not new, yet racial disparities persist (Leboy and Madden, 2012; Whittaker and Montgomery, 2014; National Center for Science and Engineering Statistics, 2021). Such a complex and entrenched problem requires multifaceted, adaptive responses. We theorized a NIC organizational structure would provide better understandings of the complex and chronic problem of the underrepresentation of Black, Latinx, and Native scholars in STEM faculty careers than traditional multi-institution organizational structures. A NIC brings together collective expertise and provides time and space to learn about varied local contexts, so each campus can adapt its infrastructure and interventions to its different contexts and local partnerships. Interventions developed and adapted by the NIC described in this article range from faculty member attitudes and behaviors, identity development and self-efficacy of AGEP scholars, inclusive climate of the lab or research group where students spend much of their time, to departmental, college, and university climate.

Previous NICs in educational settings (typically K-12) have strong central organization through their district or charter network (LeMahieu et al., 2017). However, such centrally organized and tightly coupled systems are rare among higher education institutions. Indeed, a distinctive feature of this NIC is its diverse institutional composition. Our member institutions were all part of the Center for the Integration of Research, Teaching, and Learning (CIRTL), a national consortium committed to inclusive STEM higher education. All CIRTL AGEP NIC member universities are high or very high United States research doctoral universities, yet they are structurally and culturally quite different. They include both public and private, predominantly white institutions and minority serving institutions (a historically Black university and a Hispanic-serving institution), and several land grant institutions located across eight states and Washington, DC, United States.

Our NIC’s dispersed membership was particularly valuable for gathering information about different initiatives and their potential at institutions with varied contexts, missions, and cultures. Institutional representatives to the NIC held different positions within their institutions, including faculty, staff, administrators, and graduate students. The varied positionalities of individual members was a strength, offering important and complementary expertise for the conceptual and practical work at the NIC-level and on our campuses. Each individual had access to varied levers of change, which influenced their perspectives and contributions to the NIC, and led to a renewed focus of the NIC on building local infrastructure. As such, each institution chose its own interventions to be responsive to campus needs.

The authors of this community-case study are all active participants in the NIC. Three are graduate students, six are professional staff members in a graduate school, three are professional staff members in a teaching and learning center, two are faculty members in STEM, three are faculty members in higher education, four are assistant or associate deans in the graduate school, and three are deans of a graduate school. Graduate students received assistantships, faculty received some summer salary, while administrators on 12-month appointments were not paid any additional amount for NIC work.

In the following sections, we share our initial steps in forming the NIC, work to improve and create a more inclusive NIC, the interventions we implemented on different campuses, and the lessons we have learned along the way. We drew on evaluation data, including bi-weekly and annual meeting observations, interviews, and surveys, as well as members’ reflections to inform this community case study.

## Initial Steps in Forming Our Networked Improvement Community

Carnegie describes four recommended parts for a NIC as a well-specified aim, a deep understanding of the problem, the utilization of improvement science methods, and a focus on accelerating interventions (McKay, 2017). In the initial stages of our CIRTL AGEP NIC, we worked on these four components.

### Well-Specified Aim

The group coalesced during the writing period for the grant around the main goal of the AGEP Request for Proposals, which is to increase participation of those from backgrounds historically marginalized in STEM faculty careers. The aim for our project needed to be narrowed in order to be achievable within the 5-year period of the grant and be within the scope of participating universities. After several rounds of discussion, we defined the aim to be:

“Increase the number of Ph.D. candidates/postdocs who are interested in faculty careers by 50%.”

This had several advantages: (a) Interest in faculty careers was something we could influence; (b) Such interest is a logical prerequisite for students and postdocs applying for faculty positions; (c) Prior research indicates interest in faculty careers drops significantly for those from historically marginalized backgrounds (Gibbs et al., 2014); and (d) It did not depend on factors outside the project’s scope such as university hiring committees. In a pilot survey of some NIC universities, we defined interest in a faculty career as “interested” or “strongly interested” in a faculty career in either a 4-year university or college with a research mission, a teaching mission, or a combination, or a faculty career in a 2-year or community college. Final data on students’ interest in faculty careers have not been collected at this point in the project.

### Understanding the Problem

**FIGURE 1:**
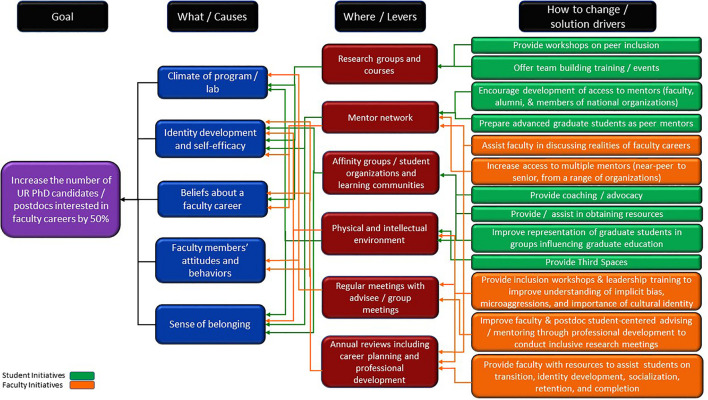
Driver diagram for the CIRTL AGEP NIC.

The driver diagram (Figure 1) represents our NIC’s understanding of the main influences or primary drivers for reaching the aim (or goal). These are shown progressively to the right of our goal. On the far right are initiatives or projects that could help make progress on the drivers. If progress was made on each driver, we would achieve the project’s aim.

Our CIRTL AGEP NIC built the driver diagram through iterative, collaborative, and consensus decision-making processes over several months with revisions in subsequent years as the project progressed and matured. The CIRTL AGEP NIC built consensus on the five primary drivers (“What/Causes” blue boxes) using evidence-based research to reach a common understanding of the importance, value, and meaning of each driver: the climate of program/lab; students’ identity development and self-identity; beliefs about a faculty career; faculty members’ attitudes and behaviors toward the student; and the students’ sense of belonging. Moving to the right of the driver diagram, the “Where/Levers” column (maroon boxes) identifies locations or times in the system where these drivers can be affected through changes in norms, policies, or structures. The final column lists possible interventions that could change or improve specific practices that occur in these locations/times. In this diagram, the green boxes represent student-focused initiatives and orange boxes are faculty-focused initiatives. Reading the driver diagram from right to left, the improvement hypothesis is that by implementing these interventions, we improve experiences of future faculty from historically marginalized backgrounds at these locations/times, which improves the five primary drivers, which then helps reach the goal of increasing interest in faculty careers among the AGEP population.

There are more interventions on the right-hand side of our driver diagram than can be implemented on any given campus by any one team. The diversity of institutions in our NIC, the different contexts, and the variety of local partners and resources mean certain interventions are more viable on some campuses than others. The list of possible interventions is a multi-layered approach to achieving our goal, with each institution implementing select interventions deemed most appropriate for their campus and students, most often in collaboration with other campus units not directly engaged in the NIC. Select interventions are described later in this paper. Putting the driver diagram into practice gave us the opportunity to try multiple approaches toward achieving the same goal, which facilitated critical learning within the NIC. In some cases, interventions started on one campus were adopted or modified on one or more other NIC campuses, but no campus implemented all interventions and some interventions were attempted by only one campus.

### Improvement Science

The NIC theory of improvement hypothesizes that if the project can improve the drivers, we will make progress toward our target goal. We constructed a comprehensive survey instrument containing questions related to each of our five primary drivers. Although the survey provided a partial initial baseline for the project, it was not useful for improvement science. Only five of nine universities (the tenth university’s role was evaluation of the 9-institution NIC) were able to distribute the survey to their graduate students, postdocs, and/or faculty; the other universities relied on data from similar surveys already established at their institutions. As the project progressed, concerns were expressed that the survey was too long and was not validated appropriately, so it was not re-administered after the initial data collection.

Formative evaluations of campus interventions that collected information about the impact workshops had on participants proved more useful. Questions such as “when were you most or least engaged?” were used to improve workshops (Brookfield, 1995). These evaluations were not identical across campuses (although that is a recommended practice for NICs) because workshops were tailored to each campus and therefore, different in content and structure. In some cases, evaluation instruments already in place on a campus or widely used by campus partners were used.

### Accelerate Interventions

Each participating university worked toward our shared goal by utilizing different approaches, e.g., improving inclusiveness of the climate in departments and research groups, holistic admissions processes, peer mentoring, and improved advising. They implemented local interventions focused on different portions of the driver diagram such as faculty member attitudes and behaviors, identity development and self-efficacy of AGEP scholars, and the inclusive climate of the lab, research group, or department. In all cases, campus interventions were joint projects with local campus partners, e.g., workshops on climate were done in conjunction with department, college, and university initiatives.

Some sharing of workshops between NIC campuses occurred, most notably on holistic graduate admissions. Selection and design of interventions were influenced by the varying local contexts and the interests of partners on each campus. As the CIRTL AGEP NIC developed, the local context, existing or potential on-campus partnerships, and the extent of local capacity all became more important factors than sharing workshop materials produced at different NIC institutions. As a result, instead of sharing and adopting multi-campus interventions, our NIC focused more on strategies to identify and foster local partnerships and infrastructure that might be most effective for introducing and accelerating local interventions appropriate to each campus context. By partnering with local units on a campus, the interventions differed across the NIC.

Finally, we learned that analyzing and addressing failed strategies is as important to a healthy NIC as sharing the learning from successful interventions. These discussions required a level of trust among members and willingness to share concerns, biases, and institutional challenges often kept silent in cross-university funded projects. Trust-building discussions were intentionally added to annual and bi-weekly meetings of the NIC. As a result, deeper understanding emerged from sharing experiences in this way, often facilitating campus efforts more quickly than when partners only share context-specific “best practices.”

## Continuous Improvement in Our Networked Improvement Community

The formation of our NIC relied on pre-existing relationships among individuals who had come to know one another through their interactions via the CIRTL Network, leading to assumptions about how we would work together based on past patterns rather than making explicit how the NIC would establish norms, understand common processes, and manage changing relationships. However, both the goals and the structure of the NIC were different from the prior context where these relationships started. During the initial period of our NIC, it became apparent to evaluators that the people and institutions involved varied in: (1) Available resources, (2) Capacity of people who could work on the project, and (3) Positions that NIC members held on their campuses, which ranged from graduate students to graduate deans, STEM faculty to social science faculty, and staff working on diversity, equity, and inclusion (DEI) initiatives. Results from our evaluation team indicated that not all voices felt heard, and some members felt they could not raise concerns. Some perceived there to be dominant and secondary voices/institutions.

As we struggled to create a more inclusive NIC, we examined our leadership model. Innovation, efficiency, collaboration, and transformation are sacrificed when all voices are not heard and valued. How could we structure our group differently from traditional collaborative projects? Was there a different paradigm? To reduce traditional hierarchies, we adhered to the basic tenets of inclusion and equity.

We found the single Principal Investigator (PI) or PI group model was not fully serving the NIC’s evolving values and aspirations, even though the funding source for our NIC project required identification of and responsibilities from designated PIs. Our NIC sought to avoid break-away affinity groups, dominance of certain voices, backroom conversations/alliances with corresponding lack of transparency, and equivocation. There may be safety and comfort (for some) in the known PI structure but it can present challenges to inclusive leadership. During these discussions, there was a confluence of two additional changes: (1) Some individuals and an institutional member left the NIC and (2) The NIC moved to a more collaborative leadership model (Routhieaux, 2015) adopting rotating responsibility for setting meeting agendas, sharing meeting facilitation, and continued use of a more sociocratic decision-making process. Emergence of new voices and energies investing in the process served as a key indicator that our strategy for increasing inclusivity was working.

For example, historically Black colleges and universities (HBCUs) lead the way in producing Black undergraduates who enter and succeed in STEM doctoral programs (Upton and Tanenbaum, 2014), yet these institutions are often relegated to subordinate roles in multi-institution consortia. Their presence at the table when discussing graduate education can be seen as tokenism rather than valued representation. In our evolving CIRTL AGEP inclusive leadership approach, Howard University took on a larger role and shared their successful mentor model across the new NIC.

Moving toward a more inclusive NIC also meant we increased discussions about the context of each university, the positionality of NIC members at their university, and their local partners, conversations that had not happened since early in the grant. Local infrastructure, partnerships, and capacity to implement change became paramount. By providing more opportunities for people to share their context and discuss possible solutions that could be adapted from other campuses, this NIC model accelerated change on multiple campuses.

We found restructuring our NIC with a focus on inclusivity turned the challenge of a variety of institutions and contexts into a strength of diversity. We learned from each other, especially how to work with different partners on each campus and build organizational capacity. In a few instances, adapted interventions provided diverse contexts to test and led to stronger solutions on each campus.

**FIGURE 2:**
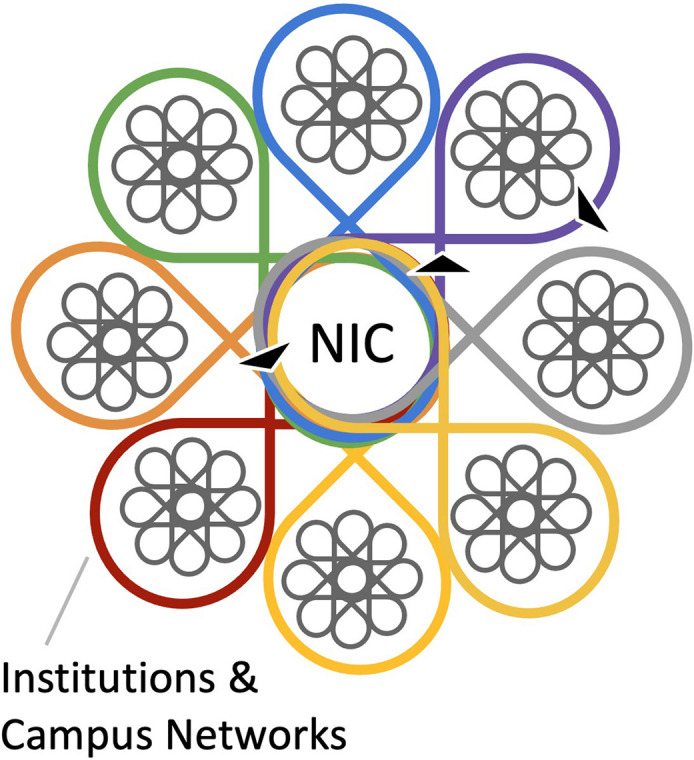
A schematic representation of a NIC that recognizes each campus’ context, shares possible solutions and ideas for infrastructure, and hence accelerates change on each campus. The colors represent different NIC institutions. The arrows represent the information flow between institutional members and the NIC, and from the NIC to institutional members.

Our CIRTL AGEP NIC model is represented in Figure 2. Each campus is shown as implementing interventions in their own context and with key campus partners, while at the same time the members of the NIC seek to connect with each other equitably across the network to share what is working and what challenges they may have.

An unresolved issue is the necessary infrastructure underlying this NIC model. Our project did not originally budget for a project manager or person with responsibility for facilitating the administrative needs of the NIC. Doing so would have undoubtedly helped with various organizational tasks, such as ensuring meeting agendas were set and communicated in a timely manner, attending to logistics of hosting in-person meetings, providing technical support for facilitating virtual meetings, tracking NIC activities, and coordinating outreach efforts. However, having a single project manager could have given more perceived influence to a single voice, potentially decreasing the NIC’s inclusivity.

In Table 1, we compare principles and purposes of a traditional NIC with our CIRTL AGEP NIC. The main differences in our more inclusive NIC are explicit attention to shared leadership, understanding and respect for different contexts and local partners, positionality and capacity of the organizations, and the focus on infrastructure in addition to interventions.

**TABLE 1:** A comparison between the principles of a traditional (Carnegie) NIC and our CIRTL AGEP NIC.

**Traditional NIC (Carnegie NIC)**	**CIRTL AGEP NIC**
Well-specified aim	Well-specified aim
Understanding the problem and how to address it	Understanding the systems in which the problem is located, including the variety of contexts, local partnerships, and different organizations
Improvement science methods, such as Plan, Do, Study, Act (PDSA) cycles	Incorporating data that reflect different campus contexts and varied analytical approaches while providing utility to the collective
Leadership models are not specified	Culture and practice of shared leadership in determining questions to be addressed and actions to take, centered around inclusivity in our practices
Accelerate interventions	Accelerate local partnerships, infrastructure and interventions

We continue to interrogate our CIRTL AGEP NIC model, and what is required to incubate and accelerate transformation toward equity, diversity and inclusion. As a result of these changes from a traditional NIC, this more inclusive NIC structure has required significant engagement within the group, more intensive than traditional multi-institution organizational structures. This structure has also highlighted the value of building intentional, trusting relationships and those relationships’ role in advancing DEI work.

## Changes on Our Networked Improvement Community Campuses

Throughout development of the CIRTL AGEP NIC, from the initial steps following the four-part NIC model to the more inclusive NIC model, members of the NIC have been implementing changes on each campus to make progress on project goals. A sample of these interventions is listed in Table 2. Where more than one university is listed next to an intervention, members of the NIC used an intervention started on one campus and adapted it to a new context. Differences in local contexts and the need to work with local partners required that different interventions be implemented across the NIC.

**TABLE 2:** Sample of interventions and their alignment with CIRTL AGEP’s driver diagram.

**Driver**	**Solution drivers**	**Examples**
Faculty member attitudes and behaviors	Strengthen understanding of DEI	• My Voice My Story (Cornell University, University of Georgia, University of Maryland) • Inclusion workshops (University at Buffalo, Howard University, Iowa State University) • Inclusive teaching (Northwestern University, The University of Texas at Arlington)
	Improve admissions, advising, and mentoring	• Holistic admissions (University of Georgia, University of Maryland, Cornell University) • Faculty advising and mentoring (University of Maryland, University of Georgia, Cornell University)
Identity development and self-efficacy	Increase peer support and professional development	• Peer mentoring (Howard University, The University of Texas at Arlington) • NextGen Professors (Cornell University) • Formation of a Graduate Student of Color Association (University at Buffalo)
Climate of lab/research group	Work with DEI partners and other offices on campus	• Mini-grants for department or college DEI initiatives (Boston University, Northwestern University) • Diversity partners in colleges (Cornell University, Iowa State University, University at Buffalo, The University of Texas at Arlington)

Interventions listed in Table 2 are categorized by the different drivers of our driver diagram (Figure 1). Campuses worked with local partners to strengthen faculty members’ understanding of DEI concepts and to improve the processes of admissions, advising, and mentoring. Other campuses implemented peer support and professional development for scholars from historically marginalized backgrounds to increase their interest in and preparation for faculty careers. A third group of campuses focused their interventions on the climate of labs and research groups through mini-grant programs or by partnering with ongoing DEI work in departments or colleges.

• *My Voice, My Story* sessions pair video monologues – constructed from experiences of graduate students – with facilitated discussions. The primary objectives are to utilize the power of narrative to achieve greater understanding of the lived experiences of graduate and professional students, share stories that frequently go untold, and develop strategies on how to create more inclusive and supportive research and learning environments (Cornell University, 2021a).
• Inclusion workshops, separately offered for graduate students and faculty, promote more in-depth knowledge and understanding of privilege, marginalization, microaggressions, implicit bias, and structural racism. These workshops can be run by content experts or in a train-the-trainer mode. In the latter, approximately 1.5 days of training helps prepare faculty and graduate student facilitators to run inclusion workshops.
• The inclusive teaching initiatives center department conversations on diversity, equity and inclusion as a core part of faculty’s work. Departments adapt a set of inclusive teaching principles to their context within a university-wide framework.
• Holistic admissions adapts the framework developed by Posselt (2016). Programs reflect on what strengths and attributes they are really looking for in graduate students and find ways to gather that information when a student applies. Admission decisions draw on this broader range of information, and as a result some programs elect to not require GRE scores. Others ask for both an academic statement of purpose and a personal statement with a diversity focus from all applicants.
• Professional development for faculty on advising and mentoring graduate students draws on several resources, e.g., Center for the Improvement of Mentored Experiences in Research (CIMER Center for the Improvement of Mentored Experiences in Research, n.d.; see also Branchaw et al., 2020) and Sloan University Centers for Exemplary Mentoring (Alfred Sloan Foundation, 2021). They feature an inclusive, student-centered framework, with discussions on understanding both one’s own and students’ social identities, jointly agreeing to expectations, communication, empowerment, and faculty support for the broad range of careers a student may be interested in.
• Peer mentoring provides an opportunity for new graduate students from AGEP populations to learn from more experienced graduate students. Training is provided for the mentors, as well as a suggested structure for the conversations and how to develop the mentoring relationship. Community is built by gathering the mentors and mentees together during the first year. This program helps new students transition and addresses many of the challenges that students are experiencing.
• NextGen Professors is a career-development program focused on preparing graduate students and postdocs for faculty careers across institutional types. The primary audience is doctoral students (in year three or beyond) and postdocs from backgrounds historically underrepresented in the professoriate, and/or those with a demonstrated commitment to advancing diversity, inclusion, access, and equity in academia (Cornell University, 2021b).
• The formation of a Graduate Student of Color group provides space for students to share their issues and experiences, support each other, and come together as one voice articulating their needs and requests to improve their education.
• Mini-grants aim to improve the local climate in departments and research groups by allowing them to drive their own local change. Interested departments or colleges apply to a broad request-for-proposals with diversity and inclusion initiatives that best address their local context.
• Partnering with ongoing DEI initiatives being run by local campus partners serves two purposes: (1) It connects graduate-level work with university-wide initiatives, and (2) Helps sustain the work past the life of the NIC by building capacity and local infrastructure.

## Recommendations

We offer several recommendations for consideration by future NICs as they plan their formation and work to establish a culture of equity and engagement.

1. NICs should use an inclusive, shared leadership model. Welcoming all voices contributes to innovation, efficiency, collaboration, and transformation. Perhaps because of initial relationships and ways of interacting established before the start of our NIC, participants brought different assumptions and expectations to the group regarding how these processes would operate, leading at times to awkwardness, fractured relationships, and institutional and individual departures from the NIC. After changing to a more shared leadership model, we saw the emergence of new voices and energies investing in the process. In our CIRTL AGEP NIC, our shared leadership model helped equalize voices independent of the institutional prestige and the professional position of the member. Shared leadership in collaborations like a NIC can help reduce hierarchies and the potential for exclusion based on previous relationships, while also helping build upon those relationships in the new context.

2. NICs should define membership in the NIC, including the associated responsibilities and benefits of membership, as well as how member contributions will be recognized and honored, and should devote time to building an inclusive, shared NIC culture. In NICs, membership is a combination of institution and individual. With new individuals in the NIC as well as some continuing from CIRTL came a new culture that needed attention and time to build equitable norms and expectations about individual roles and shared leadership. Additionally, over the scope of a multi-year NIC project, individuals within an institution change. Welcoming and onboarding efforts are needed to bring new individuals (even from existing institutions) into the NIC fold, but also important, the NIC culture must be amenable to adapting to its own changing composition and the new ideas that come with new participants. As the Carnegie Foundation notes, “A well conceived and supported NIC builds trusting relationships that allow members to respect the contributions that each brings to the collective effort” (LeMahieu, 2015, p. 8).

3. NICs should have an organizational structure agreed to by all members. Project funding should include support for maintaining and scaffolding the organizational structure, e.g., a project manager. Careful coordination is required for NICs to work well (LeMahieu, 2015). Our NIC relied on the good will and largely uncompensated efforts of individuals within the NIC to volunteer to take on various administrative and organizational tasks, such as setting meeting agendas, hosting in-person meetings, facilitating virtual meetings, tracking NIC activities, coordinating outreach efforts, etc. Over time, willingness and/or ability to volunteer for these duties diminished. Having a funded project manager for the duration of the project with well-defined duties and responsibilities, could benefit the group’s functioning and productivity within a shared leadership structure, including a focus on strengthening inclusive practices.

4. NICs should articulate mechanisms for conflict resolution, decision-making, data management, onboarding and offboarding, and other processes necessary to provide a transparent, respectful climate required for the types of sharing and learning necessary in a well-functioning NIC. This is particularly true when the different group members are from organizations with different resources. Additionally, such mechanisms will help address the inevitable challenges of individual members changing over the course of a multi-year project.

5. NICs should wrestle with the challenge of individual members holding different positions in their respective institutions, with varied access to resources and varied levels of influence on levers of change. In some member institutions, key university administrators (e.g., academic deans) with clear budget authority were active NIC participants while in other member institutions active NIC participants were professional program staff experts in program delivery. Budgets and access to partners in each NIC institution varied widely, with some institutions having considerable budget flexibility and many willing campus partners while other institutions’ representatives worked within significant budget constraints and with few, if any, campus partners beyond their own units. At times, such disparities caused tensions within our NIC group discussions. Because grant-funded project budgets are unlikely to be able to equalize such institutional disparities, NIC members need to deal openly with these inequities and agree on productive ways to work together to the best of each institution’s and each individual’s abilities, budgets, and resources.

## Limitations

In this community-case study paper we restricted ourselves to United States-based research universities working together on a government-funded project. Some findings may not be applicable to NICs or universities in other cultures or countries. Our focus has been on the climate in STEM disciplines, so this discussion may not be fully applicable to humanities and social sciences.

## Conclusion

We began this article by summarizing the Carnegie Foundation’s four components of a NIC, including a well-specified common aim, an articulated understanding of the problem and theory of change to reach that aim, ability to engage in improvement science, and coordination to accelerate interventions toward addressing the identified problem. However, within this model, our NIC at the early stages struggled with its leadership structure, was not able to fully engage in improvement science, and benefited in only a few instances where universities partnered on common interventions. Differences in local contexts and the need to work with local partners required that different interventions be implemented across the NIC.

Intense reflection, discussion, time, and effort led to a revised, more inclusive NIC. We continue to interrogate our CIRTL AGEP NIC model and what is required to incubate and accelerate transformation in equity, diversity, and inclusion. In our NIC model, the main differences from a traditional NIC are explicit attention to shared leadership, inclusive practices, understanding and respect for different contexts, local partnerships, positionality, and capacity of different organizations in the NIC, and a renewed focus on using examples from across the NIC to learn how to support and strengthen the infrastructure and local capacity on each campus. NIC structures have much to offer those seeking to advance DEI efforts in the STEM higher education landscape, especially as a means of accelerating learning, support for improving change practices, and when the group forming and maintaining a NIC engage in their own work to create an inclusive organization.

## Data Availability Statement

The original contributions presented in the study are included in the article/supplementary material, further inquiries can be directed to the corresponding author.

## Ethics Statement

The studies involving human participants were reviewed and approved by the Iowa State University IRB. The patients/participants provided their written informed consent to participate in this study.

## Author Contributions

CN took the lead in writing this manuscript with significant contributions from BK, CM, CO, AD, KD, PS, JM, and MA. All co-authors edited and reviewed the manuscript. MA, JC, AD, CM, RM, CN, and AW did the work to change to an inclusive NIC. LC, KD, JF, BG, TH, GH, SH, TH-B, OK, BK, JM, CO, NR, PS, and AW led campus initiatives. AG, CN, and MA gave evaluation results. RM, RP, and SR gave social science input and results.

## Conflict of Interest

The authors declare that the research was conducted in the absence of any commercial or financial relationships that could be construed as a potential conflict of interest.

## Publisher’s Note

All claims expressed in this article are solely those of the authors and do not necessarily represent those of their affiliated organizations, or those of the publisher, the editors and the reviewers. Any product that may be evaluated in this article, or claim that may be made by its manufacturer, is not guaranteed or endorsed by the publisher.
